# Supplementary material for: Optogenetic control of RhoA reveals zyxin-mediated elasticity of stress fibres
Source: Nat Commun. 2017 Jun 12;8:15817. doi: 10.1038/ncomms15817 (PMC5477492; doi:10.1038/ncomms15817)
Supplement: Supplementary Information — Supplementary Figures, Supplementary Table, Supplementary Note and Supplementary References [file ncomms15817-s1.pdf]

Supplementary Figure 1

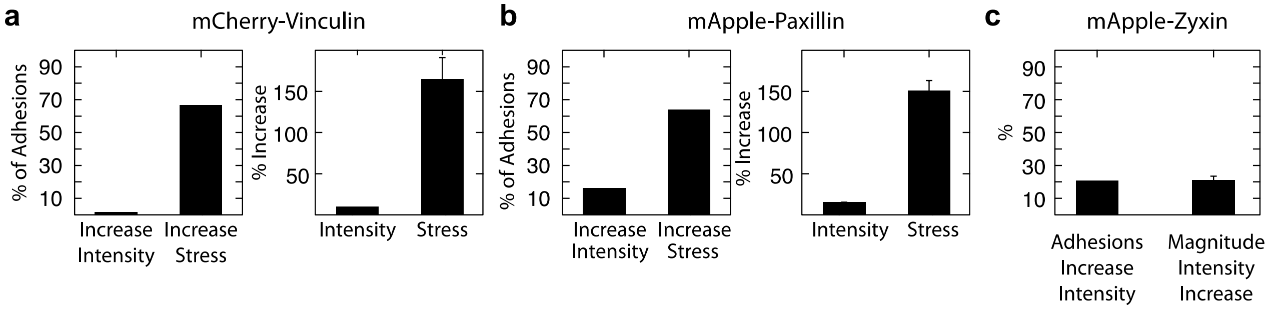

Statistics for changes in adhesion morphology during local RhoA Activation. (**a**) The percentage of adhesions that increase in vinculin intensity and applied stress during local RhoA activation, and the magnitude of the relative increase, in the cell shown in Figure 2. (**b**) The percentage of adhesions that increase in paxillin intensity and increase in applied stress during local RhoA activation, and the magnitude of the relative increase, in the cell shown in Figure S4. (**c**) The percentage of adhesions that increase in zyxin intensity, and the magnitude of that intensity increase in the cell shown in Figure 5.

Supplementary Figure 2

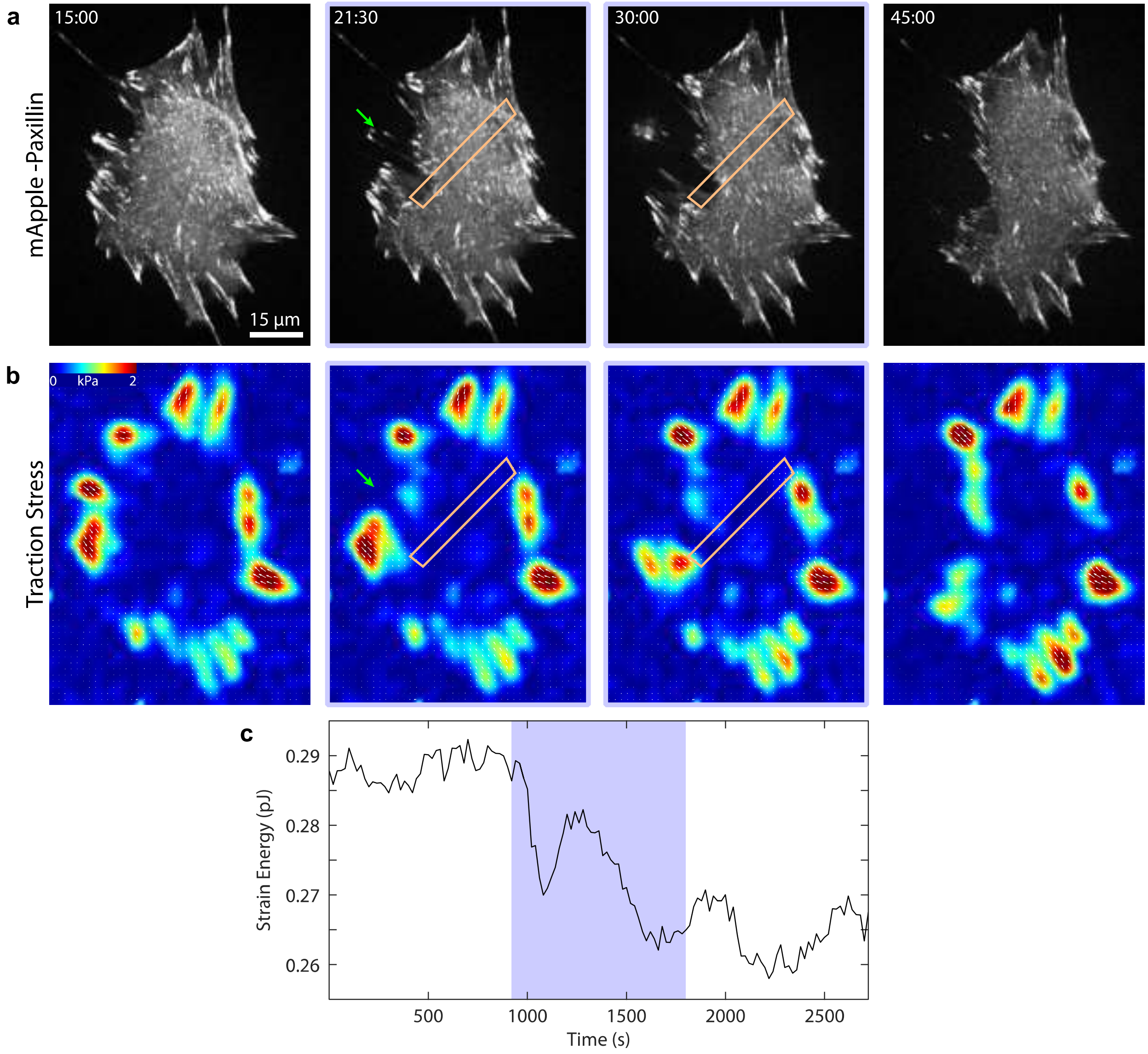

Too much local activation can result in adhesion failure. (**a**) A time series showing a cell expressing mApple-paxillin during local activation of RhoA (orange box). The green arrow illustrates the focal adhesion that ruptures upon local activation. Time is min:sec. (**b**) The corresponding traction maps for the cell shown in **a**. (**c**) The strain energy as a function of time during local RhoA activation (blue background). Upon local activation a focal adhesion rips off the substrate leading to a sudden loss in stain energy.

Supplementary Figure 3

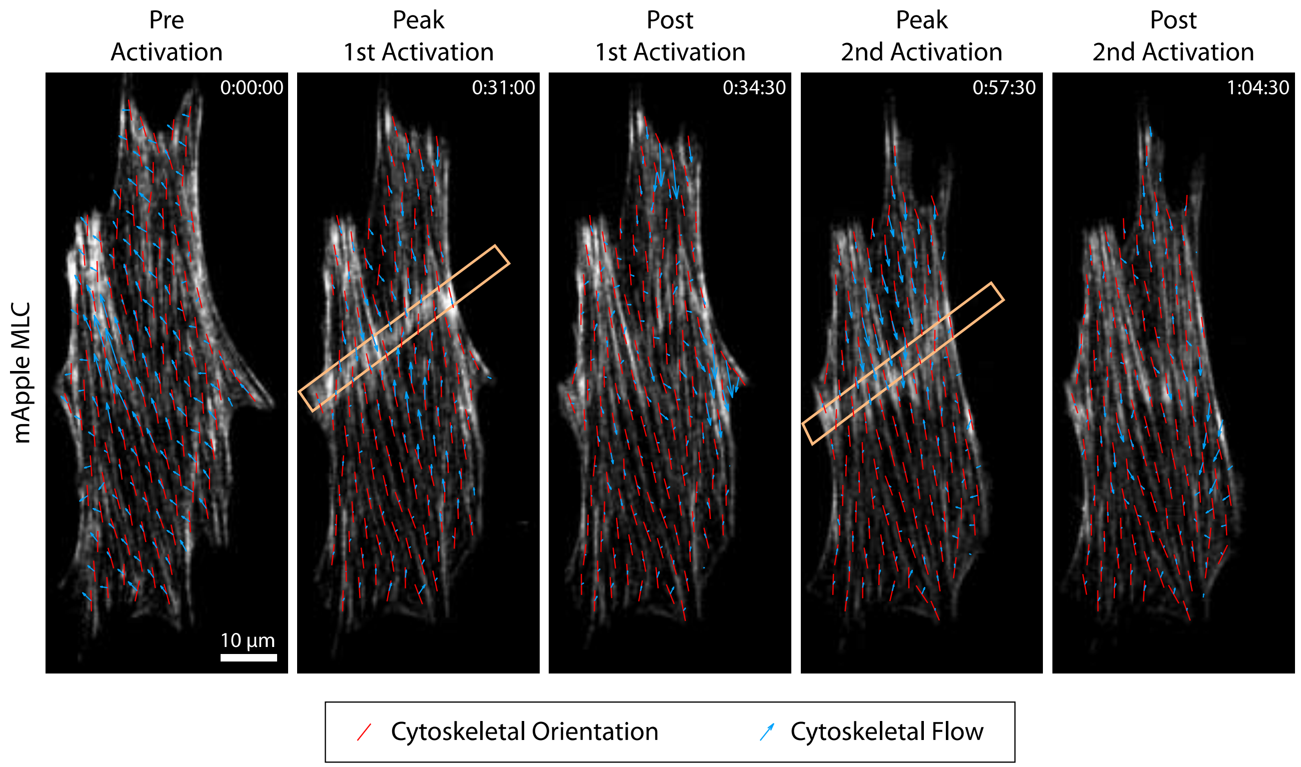

Cytoskeletal flow is primarily directed along the orientation of stress fibers. A time series of local RhoA activation (orange box) in a cell expressing mApple-MLC. The local cytoskeletal orientation is overlaid as red bars, while the local cytoskeletal flow vectors are shown in blue arrows. Time is hr:min:sec.

Supplementary Figure 4

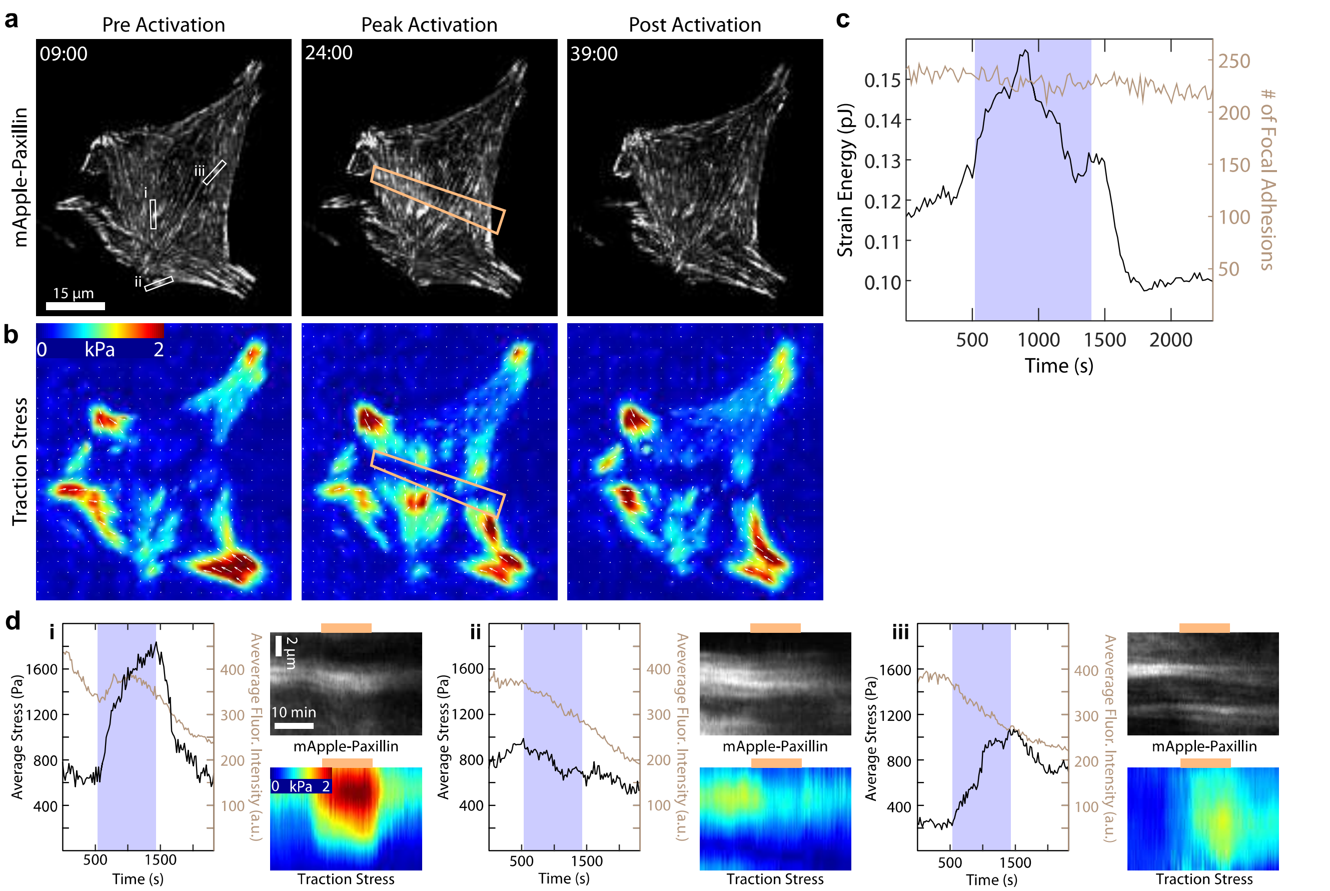

Paxillin accumulates at sites of strain along stress fibers during local activation of RhoA. (**a**) A time series of an NIH 3T3 fibroblast expressing mApple-paxillin during local activation (orange box) of RhoA. (**b**) The corresponding traction maps for the time series in **a**. (**c**) The strain energy of the cell and the number of focal adhesions during local activation (blue background). The number of adhesions stays relatively constant throughout activation. (**d**) The average stress and fluorescence intensity of the adhesions marked by white boxes in **a**. Also shown are kymographs generated by drawing a line along the long axis of the indicated adhesion.

Supplementary Figure 5

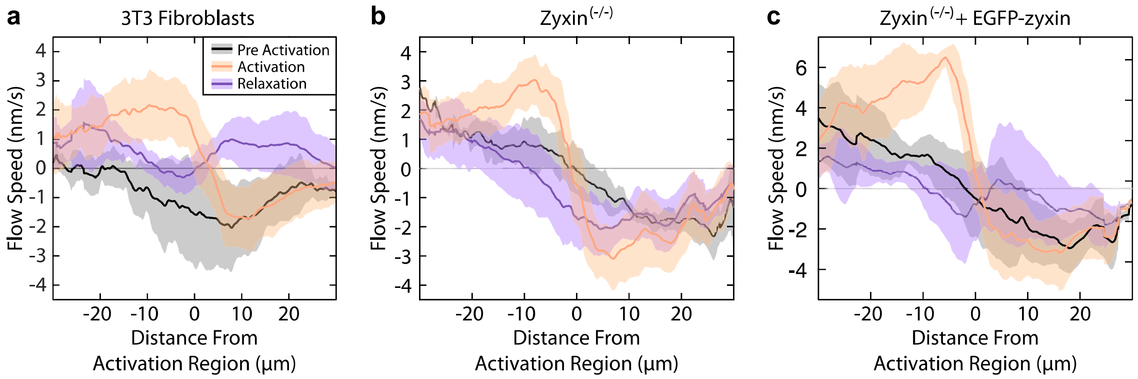

(**a**) The average flow rates along stress fibers before, during, and after local RhoA activation in NIH 3T3 fibroblasts. (**b**) The average flow rates along stress fibers before, during, and after local RhoA activation in zyxin$^{(-/-)}$ MEFs. During activation, flow still increases from both ends of the fiber toward the activation region. During relaxation, however, flow speeds return to pre-activation rates but do not exhibit any reversal of direction. (**c**) Average flow rates in zyxin$^{(-/-)}$+EGFP-zyxin MEFs. Rescue of zyxin in the knockout cells recovers the elastic flow reversal behavior seen in the 3T3s.

Supplementary Figure 6

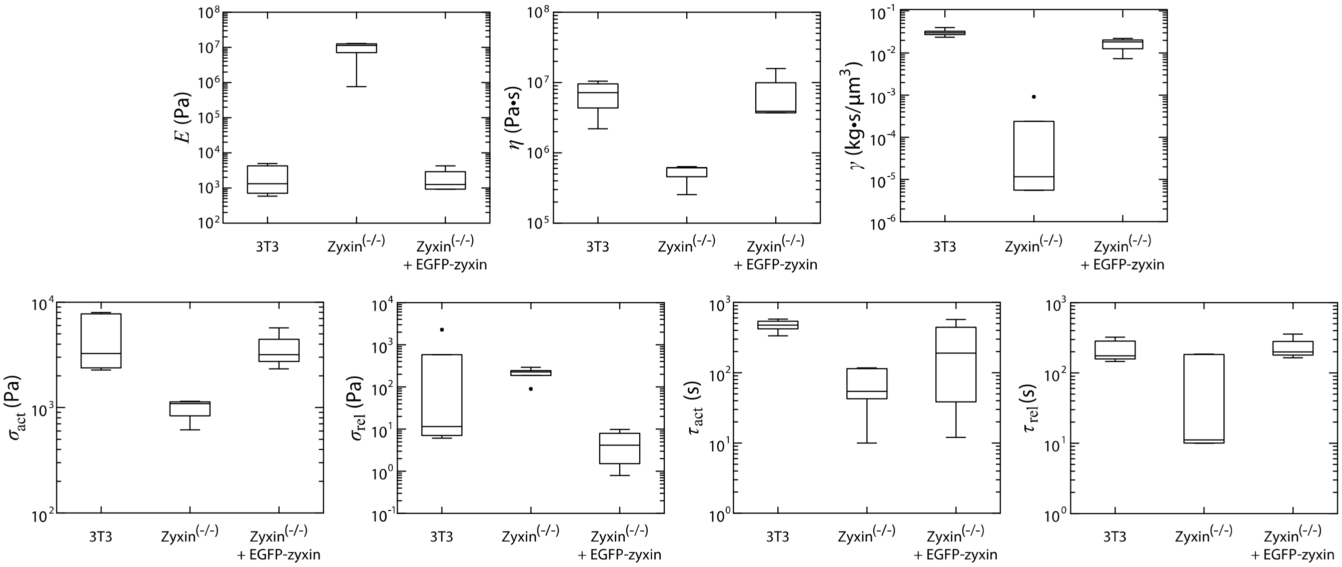

Boxplots representing the fitting parameters for the continuum model for 3T3s, zyxin$^{(-/-)}$ MEFs and zyxin$^{(-/-)}$+EGFP-zyxin MEFs. The parameters represent the elastic modulus (E), the viscosity (η), the frictional coefficient (γ), the activation stress (σ_{act}), the relaxation stress (σ_{rel}), the activation stress time constant (τ_{act}) and the relaxation time constant (τ_{rel}).

Supplementary Note

Continuum Modeling

We are looking for a simple model consistent with the following requirements:

- The model should allow flow to occur, at least on the time scale of tens of minutes, on which stress fibers flow
- It should allow for an elastic component because forces are transmitted quickly over large distances
- Flow should be driven by gradients in myosin II activity generated by local RhoA activation

The simplest model fulfilling these requirements is an *active Maxwell model*, which consists of a spring and a dashpot in parallel with an additional active stress element, which generates a time-dependent contractile stress σ_m (see red rectangle in Supplementary Note Figure 1A). An active Maxwell model is also used in the theory of active gels, which has been applied to a wide range of flow phenomena in cells and tissue (see e.g. Prost *et al.*[1] for a review).

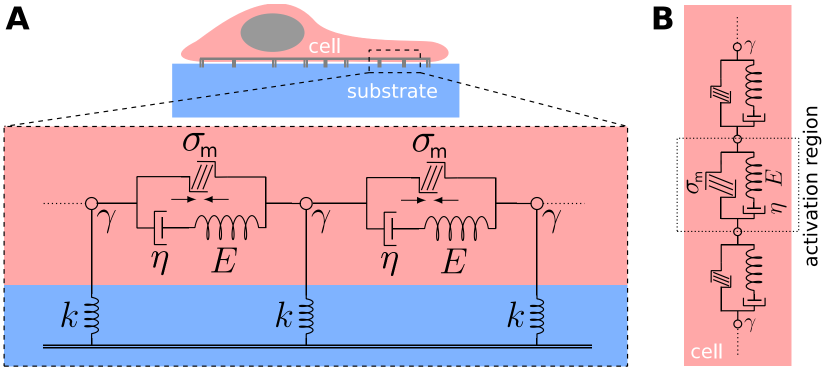

Supplementary Note Figure 1: (**A**) Effective representation of the one-dimensional cell on an elastic substrate. The cell is expressed as a myosin II motor driven viscoelastic bar which is viscoelastically connected to tits environment. In the non-activated case, each motor is assumed to exert the same contractile stress. (**B**) During activation, however, motors within the activation region pull stronger. Effectively, only the stress difference at the boundary enters the governing equations of the model.

We exclude other models for the following reasons. A solid model, e.g. a purely elastic or a Kelvin-Voigt one, has the property to relax to its initial configuration once all stresses vanish. This disagrees with the flow data in Figures 4b-d, which shows that the backflow after relaxation is significantly lower than the inflow during activation, thus the initial configuration is not reached anymore. We conclude that the

system has to have a viscous component, so that it can lose memory.

We also exclude a purely viscous model, in which both the inflow during RhoA activation and the outflow during relaxation are driven by myosin II activity. Here, the backflow after relaxation could be explained by the fact that the myosin motors outside the activation region work under stalling conditions and produce a larger effective stress during relaxation than the motors inside the activation region. However, we note that zyxin does not alter the myosin-II motor activity because otherwise there would be a significant change of the inflow dynamics during RhoA activation if zyxin is absent, which is not observed. Therefore this possibility would lead to a backflow during relaxation even in the absence of zyxin, which is not observed (compare Figure 5f). We conclude that an elastic component is needed to explain the strong backflow.

The only two-element spring-dashpot model which incorporates both an elastic and a viscous behavior is the active Maxwell model used here. In the following, we will first describe the constitutive equations of the model. We then derive the equations of motion and the finite element formulation in one and two dimensions.

One-dimensional active Maxwell Model

On a coarse-grained level, cells and tissue obey the fundamental equations of viscoelasticity[2]:

- Momentum Conservation: $\rho\,\mathbf{a}(\mathbf{x},t) = \nabla\cdot\boldsymbol{\sigma}(\mathbf{x},t) + \mathbf{f}(\mathbf{x},t) \approx 0$ for tissue
- Angular momentum conservation: $\sigma_{ij} = \sigma_{ji}$
- Constitutive relation: $\sigma_{ij}\,(\epsilon_{ij}, \dot{\epsilon}_{ij}, \dots)$

Here, ρ is the mass density, $\mathbf{a}(\mathbf{x},t)$ the acceleration vector, $\boldsymbol{\sigma}(\mathbf{x},t)$ the stress tensor, $\mathbf{f}(\mathbf{x},t)$ represents external forces and $\boldsymbol{\epsilon}(\mathbf{x},t)$ the linear strain tensor

$$\epsilon_{ij} = \frac{1}{2}\left(\frac{\partial u_i}{\partial x_j} + \frac{\partial u_j}{\partial x_i}\right), \#(1.1)$$

with u_i being the components of the displacement vector.

The constitutive equation we used for the active Maxwell model reads

$$\tau \frac{\partial \sigma_{ij}}{\partial t} + \sigma_{ij} = \eta \frac{d}{dt}\left(\lambda \delta_{ij}(\epsilon - \epsilon_{\mathrm{act}})_{kk} + 2\mu(\epsilon - \epsilon_{\mathrm{act}})_{ij}\right), \#(1.2)$$

with the Young's modulus E, the viscosity η, the Maxwell time constant $\tau = {}^{\eta}/_{E}$, the Lamé coefficients λ and μ, and the active strain rate $\dot{\epsilon}_{\mathrm{act}}$ of the motors.

During the experiments, the cytoskeleton experiences a Stokes' law friction γ with the surrounding cytoplasm. Cells on elastic substrates are, in addition, elastically coupled to their environment, which is represented by a number density N of springs with an effective spring constant k arranged in series with the Stokes' elements (see blue rectangle in Supplementary Note Figure 1A):

$$\nabla \cdot \boldsymbol{\sigma}(\mathbf{x}, t) = \gamma \mathbf{v}_{\gamma}(\mathbf{x}, t) \#(1.3)$$

$$\nabla \cdot \boldsymbol{\sigma}(\mathbf{x}, t) = kN\mathbf{u}_{k}(\mathbf{x}, t) \#(1.4)$$

Here, $\mathbf{u}_k(\mathbf{x}, t)$ and $\mathbf{v}_\gamma(\mathbf{x}, t) = \dot{\mathbf{u}}_\gamma(\mathbf{x}, t)$ are the displacement of the coupling springs and velocity of the coupling Stokes' elements, respectively. We denote $\mathbf{u} = \mathbf{u}_k + \mathbf{u}_\gamma$ as the total displacement. Note that the two terms on the right would add up for a model with parallel elements. Here however we have serial elements and therefore two equations involving the same force (which in turn is the derivative of the stress).

In the following, we will derive the PDE which governs the evolution of the tissue by combining the constitutive relation (Equation 1.2) and the momentum conservation (Equation 1.3). We consider a one-dimensional bar of width 2L, representing the cellular cytoskeleton, which undergoes an isotropic contraction. The only non-zero component of the constitutive relation (Equation 1.2) is

$$\tau \frac{\partial \sigma_{xx}}{\partial t} + \sigma_{xx} = (\lambda + 2\mu)\eta \frac{\partial(\epsilon_x - \epsilon_{\mathrm{act}})}{\partial t}. \#(1.5)$$

We will skip the indices of σ_{xx} and ϵ_x in the following.

In order to obtain the PDE which governs the evolution of the tissue, we first calculate the derivative of Equation 1.5 with respect to space x and the derivative of the momentum conservation (see Equation 1.3) with respect to time t and obtain

$$\tau \frac{\partial^2 \sigma}{\partial x \partial t} + \frac{\partial \sigma}{\partial x} = (\lambda + 2\mu)\eta \frac{\partial^2(\epsilon - \epsilon_{\mathrm{act}})}{\partial t \partial x} = (\lambda + 2\mu)\eta \frac{\partial^3 u}{\partial t \partial x^2} - \frac{\partial \sigma_m}{\partial x}, \#(1.6)$$

and

$$\frac{\partial^2 \sigma}{\partial x \partial t} = \gamma \frac{\partial^2 u_\gamma}{\partial t^2}\ , \#(1.7)$$

$$\frac{\partial^2 \sigma}{\partial x \partial t} = kN \frac{\partial u_k}{\partial t}\ . \#(1.8)$$

In Equation 1.6, we assumed a linear relation between the motor stress σ_m and the active strain rate ϵ_{act}, which is a plausible approximation of the concave force-velocity relation measured by Hill[3]. A linearization of the law, following e.g. the work of Besser et al.[4], leads to a much simpler mathematical treatment and yields rather similar qualitative results. Regarding the distribution of stresses σ_m along the viscoelastic bar, we make the simple assumption, that σ_m stays constant along the bar. Thus, the derivative $\partial \sigma_m / \partial x$ vanishes along the whole bar. The only position at which $\partial \sigma_m / \partial x$ does not vanish is the boundary between the activation and the outer region during RhoA activation (see Supplementary Note Figure 1B). Therefore, $\partial \sigma_m / \partial x$ can be skipped in Equation 1.5 and later introduced as a Neumann boundary condition (see Equation 1.17).

We can now insert Equations 1.3 and 1.7 into 1.6 with the aim to eliminate $\partial \sigma / \partial x$ and $\partial^2 \sigma / \partial x \partial t$, which yields the evolution equation

$$(\lambda + 2\mu)\eta \frac{\partial^3 u}{\partial t \partial x^2} = \tau\gamma \frac{\partial^2 u_\gamma}{\partial t^2} + \gamma \frac{\partial u_\gamma}{\partial t}\ , \#(1.9)$$

$$(\lambda + 2\mu)\eta \frac{\partial^3 u}{\partial t \partial x^2} = \tau kN \frac{\partial u_k}{\partial t} + kN u_k\ , \#(1.10)$$

or, in a rescaled version,

$$\tau \frac{\partial^3 u}{\partial t \partial x^2} = \tau \tilde{\gamma} \frac{\partial^2 u_\gamma}{\partial t^2} + \tilde{\gamma} \frac{\partial u_\gamma}{\partial t}\ , \#(1.11)$$

$$\tau \frac{\partial^3 u}{\partial t \partial x^2} = \frac{\tau}{l_{\mathrm{p}}^2} \frac{\partial u_k}{\partial t} + \tilde{\gamma} \frac{u_k}{l_{\mathrm{p}}^2}\ , \#(1.12)$$

with the localization or penetration length[5]

$$l_{\mathrm{p}} = \sqrt{\frac{(\lambda + 2\mu)E}{kN}}\ , \#(1.13)$$

and

$$\tilde{\gamma} = \frac{\gamma}{(\lambda + 2\mu)E}\ . \#(1.14)$$

At the beginning of the simulation, we assume that the displacement and the strain in the cell is zero, which leads to the initial conditions

$$u(x, t = 0) \;=\; 0\#(1.15)$$

$$\frac{\partial u(x, t = 0)}{\partial x} \;=\; 0\,.\#(1.16)$$

For all simulations of the cell both on the elastic substrate and on glass, we assume free boundaries. Fixed boundaries would give rise to an increase of the slope of the paths of the kymographs towards the activation region during RhoA activation, which is not observed (compare Figure 4f). From the biological perspective, by using free boundaries, we assume a formation of new stress fiber material at the focal adhesions during RhoA activation (see e.g. Oakes *et al.*[6]).

We further model the cellular contraction due to RhoA activation as a constant interior boundary stress which is applied at a distance $l_0 < L$ from the center of the layer in the Eulerian frame. This denotes the second boundary condition

$$\sigma(x = \pm l_0, t) = \;\mp\sigma_0(t)\,,\#(1.17)$$

with a typical stress profile in Supplementary Note Figure 2. The one-dimensional activation "area" l_0 is calculated via

$$l_0 = \sqrt{\frac{A_{\mathrm{act}}}{A_{\mathrm{cell}}}}\,,\#(1.18)$$

with A_{act} being the two-dimensional area of the activation region and A_{cell} the cell area.

We model two kinds of experiments by means of this one-dimensional model. First, we fit the evolution of the strain energy (Figure 2f), which the cell puts into an elastic substrate during its contraction. Here, we are interested in the dynamics of the springs k and, thus, we only consider the lower one of Equations 1.9. Second, we model the kymographs which emerge from the dynamics of the α-actinin and myosin-II compartments of the stress fibers (Figure 4b,f). Here, cells are adhering to a glass substrate, and thus, we are only interested in the upper one of Equations 1.9.

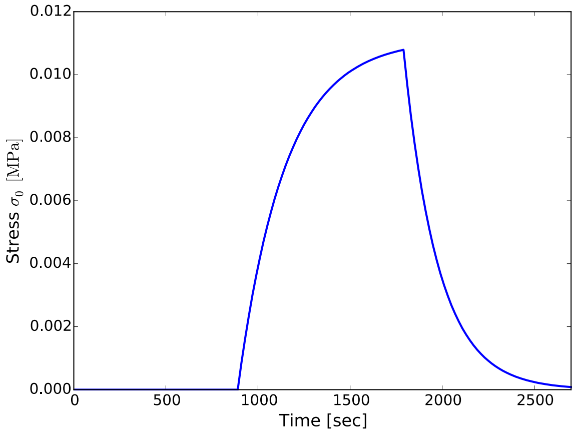

Supplementary Note Figure 2: Typical stress profile during activation of RhoA. We assume an exponential plateauing of the active stress with activation and relaxation time constants $\tau_{\rm act}$ and $\tau_{\rm rel}$.

Numerical algorithm

We use a Finite Element (FE) approach with a backward difference scheme to compute the displacement $u(x,t)$ in Equation 1.11. For this, we discretize the time $t = m\Delta t$ and space $x = i\Delta x$ with $m \in \{0, \dots, T/\Delta t\}$ and $i \in \{-I+1, \dots, I-1\}$. With the Finite Difference (FD) approximation, we have

$$u(x,t) = u_i^m \,, \#(1.19)$$

$$\frac{\partial u}{\partial t} = \frac{u_i^m - u_i^{m-1}}{\Delta t} \,,$$

$$\frac{\partial^2 u}{\partial t^2} = \frac{u_i^m + u_i^{m-2} - 2u_i^{m-1}}{(\Delta t)^2} \,.$$

The weak formulation of Equation 1.11 amounts to

$$\tau \int_\Omega \frac{\partial^3 u}{\partial t \partial x^2} v \, \mathrm{d}x = \tau\tilde{\gamma} \int_\Omega \frac{\partial^2 u_\gamma}{\partial t^2} \, v \, \mathrm{d}x + \tilde{\gamma} \int_\Omega \frac{\partial u_\gamma}{\partial t} v \, \mathrm{d}x \,, \#(1.20)$$

$$\tau \int_\Omega \frac{\partial^3 u}{\partial t \partial x^2} v \, \mathrm{d}x = \frac{\tau}{l_{\rm p}^2} \int_\Omega \frac{\partial u_k}{\partial t} \, v \, \mathrm{d}x + \frac{1}{l_{\rm p}^2} \int_\Omega u_k \, v \, \mathrm{d}x \,, \#(1.21)$$

with region Ω and test function v. We can partially integrate the term on the left-hand side which results in

$$\tau \int_\Omega \frac{\partial^3 u}{\partial t \partial x^2} v \, \mathrm{d}x = \left[\tau \frac{\partial^2 u}{\partial t \partial x} v \right]_{\partial\Omega} - \tau \int_\Omega \frac{\partial^2 u}{\partial t \partial x} \frac{\partial v}{\partial x} \mathrm{d}x \,. \#(1.22)$$

Then, the discretized version of Equation 1.20 is

$$\left[\tau \frac{\partial^2 u_i^m}{\partial t \partial x} v\right]_{\partial\Omega} - \frac{\tau}{\Delta t}\int_\Omega \left(\frac{\partial u_i^m}{\partial x} - \frac{\partial u_i^{m-1}}{\partial x}\right)\frac{\partial v}{\partial x}\mathrm{d}x = \tau\tilde{\gamma}\int_\Omega \frac{u_{\gamma,i}^m + u_{\gamma,i}^{m-2} - 2u_{\gamma,i}^{m-1}}{(\Delta t)^2} v\,\mathrm{d}x + \tilde{\gamma}\int_\Omega \frac{u_{\gamma,i}^m - u_{\gamma,i}^{m-1}}{\Delta t} v\,\mathrm{d}x\,, \#(1.23)$$

and

$$\left[\tau \frac{\partial^2 u_i^m}{\partial t \partial x} v\right]_{\partial\Omega} - \frac{\tau}{\Delta t}\int_\Omega \left(\frac{\partial u_i^m}{\partial x} - \frac{\partial u_i^{m-1}}{\partial x}\right)\frac{\partial v}{\partial x}dx = \frac{\tau}{l_\mathrm{p}^2}\int_\Omega \left(\frac{u_{k,i}^m - u_{k,i}^{m-1}}{\Delta t}\right) v\,dx + \frac{1}{l_\mathrm{p}^2}\int_\Omega u_{k,i}^m\, v\,\mathrm{d}x\,. \#(1.24)$$

The initial and boundary conditions from Equations 1.15-1.17 are implemented as

$$u_i^0 = 0$$

$$\frac{u_i^0 - u_i^{-1}}{\Delta t} = 0$$

$$u_{\pm(I-1)}^m = 0$$

$$E(\lambda + 2\mu)\left(\frac{\partial u_{\pm(j+1)}^m}{\partial x} - \frac{\partial u_{\pm(j+1)}^{m-1}}{\partial x}\right) = \sigma_j^{m+1} - \left(1 - \frac{\Delta t}{\tau}\right)\sigma_j^m\,, \#(1.25)$$

applying an active stress σ_0 at $i = \pm j$ at some time step $m = m_0$. The equations were calculated with the FE solver FEniCS[7].

Parametrization

The Equations 1.11 and 1.17 contain 10 free parameters: Elastic modulus E; Viscous modulus η; Friction coefficient γ; Maximal active stress $\sigma_{0,\max}$; Global (background) stress σ_out; Stress activation and relaxation time constants τ_act and τ_rel; Penetration length l_p; and Lamé coefficients λ and μ. We choose the two Lamé coefficients from Edwards and Schwarz[5]: $\lambda = \frac{\upsilon}{1-2\upsilon}$ with $\upsilon = 0.45$, and $\mu = 0.5$. The penetration length is calculated using an expression derived by Banerjee and Marchetti[8]:

$$l_p = \sqrt{\frac{E_\mathrm{c} h_\mathrm{c} L_\mathrm{c} l_\mathrm{sarc}}{k_\mathrm{a}} + \frac{E_\mathrm{c} h_\mathrm{c} h_\mathrm{eff}}{\pi E_\mathrm{s}}}\ \#(1.26)$$

and

$$h_\mathrm{eff}^{-1} = \frac{1}{h_\mathrm{s} 2\pi(1 + \upsilon_\mathrm{s})} + \frac{1}{L_\mathrm{c}} \#(1.27)$$

with parameters taken from Supplementary Note Table 1, yielding $l_{\mathrm{p}} = 13.6\ \mu m$. The remaining 7 parameters $E, \eta, \gamma, \sigma_{0,\mathrm{max}}, \sigma_{\mathrm{out}}, \tau_{\mathrm{act}}, \tau_{\mathrm{rel}}$ are obtained by the best fit parameter values iteratively using the Nelder-Mead method[9].

Stiffness of focal adhesion bonds k_{a}	$2.5\ \mathrm{nN}/\mu\mathrm{m}$
Lateral extent of cell L_{c}	45 µm
Length of sarcomeric subunit l_{sarc}	1 µm
Thickness of the substrate h_{s}	∞
Poisson's ratio of the substrate υ_{s}	0.5
Elastic modulus of the substrate E_{s}	25.8 kPa
Thickness of the cell h_{s}	1 µm
Approx. elastic modulus of the cell E_{c}	10 kPa

Supplementary Note Table 1: Parameters used to calculate the penetration length l_{p} from Equation 1.11

Fitting Results

We applied the numerical scheme and fitting procedure presented so far to derive numerical values to characterize the experimental results. Supplementary Note Table 2 shows the results of fitting the model to the time course of the strain energy in Figure 2f. The parameter values yield a Maxwell time constant $\tau = {}^{\eta}/_{E} \approx 5454\ s$, which is in good accordance with a recent study[10].

Elasticity E	7.37 kPa
Viscosity η	40.20 MPa•s
Stokes' friction γ	1.05 $kg/s \cdot \mu m^3$
Global (background) stress σ_{out}	0.33 kPa
Max. activation stress $\sigma_{0,\mathrm{max}}$	0.28 kPa, 1.57 kPa, 1.68 kPa
Activation time constant τ_{act}	130.12 s, 228.76 s, 130.12 s
Relaxation time constant τ_{rel}	158.29s, 158.29 s, 158.29 s

Supplementary Note Table 2: Resulting parameters after fitting the active Maxwell model to the evolution of the strain energy in Figure 2f. The three values in the lower lines refer to the three succeeding RhoA activation.

Supplementary Note Figure 3 illustrates the model fits to the kymographs presented in Figure 4. In order to be able to compare with the model, we hand-marked 10 bright paths per kymograph, each path consisting of 13 points, and introduced these paths into the fitting algorithm. We can see a qualitatively excellent agreement between model and experiment. Furthermore, Supplementary Figure

6 reveals the parameter values we can deduce from the kymographs. The resulting parameter values are in good accordance with the values we already deduced from the strain energy course in Supplementary Note Table 2. We can calculate a hydrodynamic length $\lambda = \sqrt{\eta/\gamma} \approx 15.0\ \mu m$, which is in good agreement with that of other cell types[11].

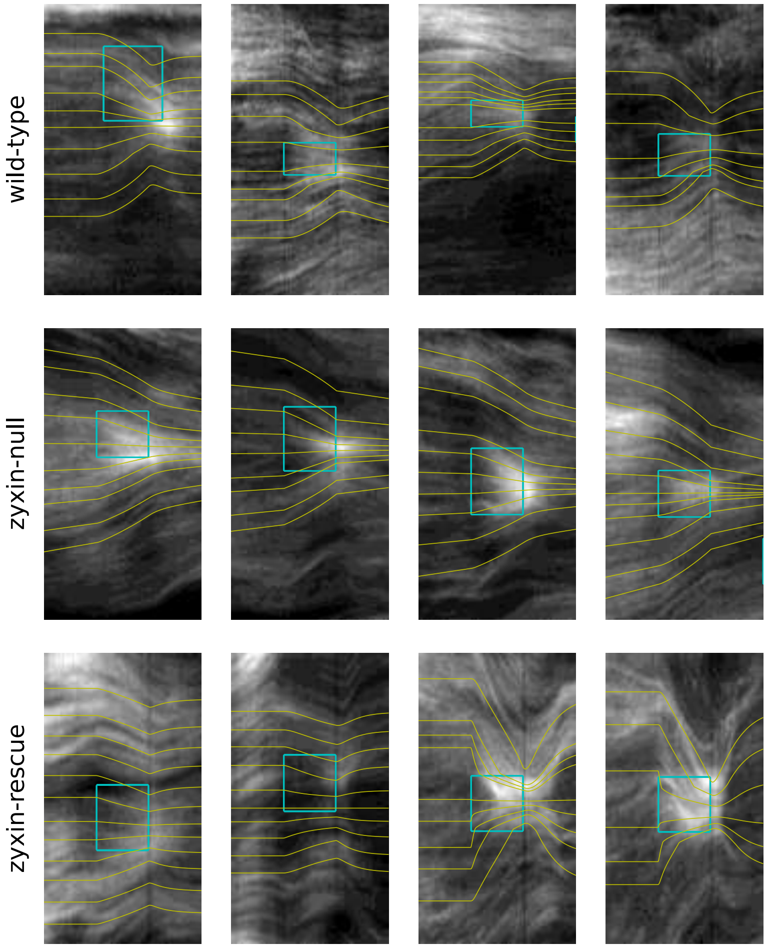

Supplementary Note Figure 3: Kymographs showing the spatiotemporal evolution of fluorescent myosin II molecules in single stress fibers during activation of RhoA, with time in horizontal and space in the vertical direction. Overlaid yellow lines show the model fits. Blue boxes illustrate the activation length (5 µm) and duration (900 s).

We can clearly see the change in magnitudes if zyxin is absent: the stress fiber becomes stiffer and more fluid, the last by both a decreased viscosity and a decreased viscous coupling to its environment. As Supplementary Note Figure 4 demonstrates, a stiffening of the stress fiber alone does not explain the experiments correctly. Thus, zyxin has an effect on both the stiffness and the viscosity of the cell.

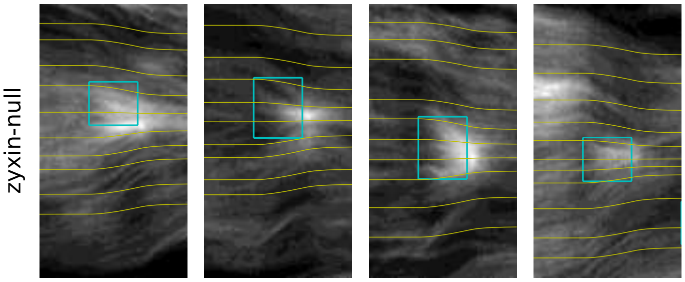

Supplementary Note Figure 4: Kymographs showing the spatiotemporal evolution of fluorescent myosin II molecules in single stress fibers during activation of RhoA for the zyxin-null experiment. In contrast to Supplementary Note Figure 3, we inserted the model parameters derived for wild-type cells and turned $E \rightarrow \infty$ (yellow lines), which, obviously, is not sufficient to describe the zyxin-null case. The system additionally has to become more fluid.

Two-dimensional active Maxwell model

We consider the two-dimensional problem of a rectangular, one-dimensionally contracting cell of Maxwell type with fixed boundary (see Supplementary Note Figure 5A). We develop the corresponding theory, following the results of the previous section, and present numerical details. The two-dimensional model is used to create a connection between the continuum model presented and the discrete model. The resulting material flow in both the continuum and discrete models is illustrated in Figure 3d.

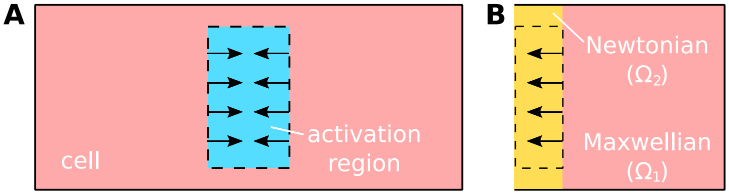

Supplementary Note Figure 5: (**A**) A two-dimensional rectangular cell, which is fixed at its boundary, experiences a linear contraction. (**B**) In order to approach the cable nature of actin filaments, the continuum simulation has to be modified: the cell is divided into a viscous (*Newtonian*) and a viscoelastic (*Maxwellian*) part. Cell material inside the activation region is modelled as viscous, non-repelling, while material outside the activation region is viscoelastic.

Theory

With regard to a comparison of a viscoelastic continuum model and a discrete cable Maxwell model, we have to modify the material as depicted in Supplementary Note Figure 5B:

• We only consider a half cell, such that the left boundary remains unfixed. Material can “vanish”

through this unspecified boundary.

• The part of the cell which is covered by the activation region is modelled as a purely viscous material in order to achieve the non-repelling character known from cables. Once material crosses the border to the activation region, it is "lost", which is equivalent to the behavior in cable Maxwell networks.

Indeed, material flow inside the activation region in the continuum model significantly differs from that of the cable Maxwell model. However, we are only interested in the flow close to the activation region, such that dynamics inside the activation region remain irrelevant for our purposes. We model the material outside the activation region, Ω_1, as a Maxwell fluid with constitutive relation

$$\tau\frac{\partial\sigma}{\partial t}+\sigma=\eta\frac{\partial}{\partial t}(2\mu\epsilon+\lambda\, tr(\epsilon)\,\mathbb{I})\,,\#(1.28)$$

while the material inside the activation region, Ω_2, is modeled as a Newtonian fluid

$$\sigma=\eta\frac{\partial}{\partial t}(2\mu\epsilon+\lambda\, tr(\epsilon)\,\mathbb{I})\,,\#(1.29)$$

with

$$\epsilon=\frac{1}{2}(\nabla\mathbf{u}+(\nabla\mathbf{u})^T)\,.\#(1.30)$$

Together with the equation of motion 1.3, we can calculate the evolution equations for both regions in analogy to above, which results in

$$\tau\gamma\ddot{\mathbf{u}}+\gamma\dot{\mathbf{u}}=\eta\frac{\partial}{\partial t}\nabla(2\mu\epsilon+\lambda\, tr(\epsilon)\,\mathbb{I})\#(1.31)$$

for region Ω_1 and

$$\gamma\dot{\mathbf{u}}=\eta\frac{\partial}{\partial t}\nabla(2\mu\epsilon+\lambda\, tr(\epsilon)\,\mathbb{I})\#(1.32)$$

for region Ω_2. We start with the initial conditions

$$\mathbf{u}(x,y,t=0)=0\#(1.33)$$

$$\frac{\partial\mathbf{u}(x,y,t=0)}{\partial x}=0\,.\#(1.34)$$

The fixed cell boundary gives rise to the conditions

$$\mathbf{u}(x=L_x,t)=0\#(1.35)$$

$$\mathbf{u}\left(x=\pm L_y,t\right)=0\,.\#(1.36)$$

Upon activation in a region of width l_0, we apply a piecewise active stress σ_0 in the *x*-direction

according to Supplementary Note Figure 6:

$$\sigma_0 = \begin{cases} -\sigma_{\max}\dfrac{l_0 - x}{l_0}, & x \le l_0 \\ 0\,, & x > l_0 \end{cases} . \#(1.37)$$

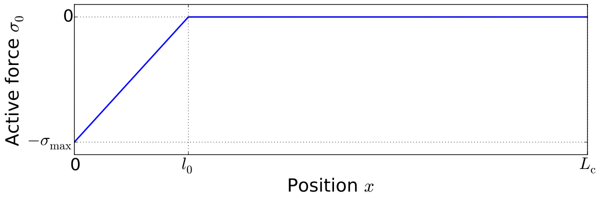

Supplementary Note Figure 6: distribution of the active stress across the cell. The active stress is zero outside of the activation region.

Numerical Algorithm

As in the previous section, we use a FE approach with a backward difference scheme to compute the displacement $\mathbf{u}(x, y, t)$ in Equations 1.31 and 1.32 with the resulting discretization in Equation 1.19.

The weak formulation of the evolution Equations 1.31 and 1.32 yields

$$\tau_1\tilde{\gamma}\int_{\Omega_1} \ddot{\mathbf{u}}\,\mathbf{v}\,\mathrm{d}\mathbf{x} + \tilde{\gamma}\int_{\Omega_1} \dot{\mathbf{u}}\,\mathbf{v}\,\mathrm{d}\mathbf{x} + \tilde{\gamma}\int_{\Omega_2} \dot{\mathbf{u}}\,\mathbf{v}\,\mathrm{d}\mathbf{x} + \int_{\Omega_1} \nabla\sigma_0\,\mathbf{v}\,\mathrm{d}\mathbf{x} + \int_{\Omega_2} \nabla\sigma_0\,\mathbf{v}\,\mathrm{d}\mathbf{x}$$
$$= \tau_1\int_{\Omega_1} \nabla(2\mu\dot{\epsilon} + \lambda\, tr(\dot{\epsilon})\,\mathbb{I})\,\mathbf{v}\,\mathrm{d}\mathbf{x} + \tau_2\int_{\Omega_2} \nabla(2\mu\dot{\epsilon} + \lambda\, tr(\dot{\epsilon})\,\mathbb{I})\,\mathbf{v}\,\mathrm{d}\mathbf{x}\ , \#(1.38)$$

with

$$\tilde{\gamma} = \frac{\gamma}{E}\ . \#(1.39)$$

We use the FE solver FEniCS to calculate the displacements in Equation 1.38[7]. The equations are solved separately for both regions with the trivial boundary condition, that the displacements resemble at the boundary between the two regions.

Discrete Model

With our model we seek to describe the interplay between elastically and viscously dominated parts of the actin cytoskeleton on the intermediate time scale of several minutes that the cell needs to rearrange its structure. As we observe the rather flat shapes of adherent cells on planar substrates, we

reduce the three-dimensional system to its two-dimensional projection. This approach significantly reduced computational complexity, while allowing insights into the role of effectively one-dimensional stress fibers (SFs) that are embedded in a higher-dimensional environment.

The majority of actin filaments are organized in distributed networks that cannot be observed by standard resolution optical microscopy. As these networks are only weakly cross-linked and subject to high turnover rates, we assume that they can be modelled as a triangulated network, whose edges obey a Maxwell type material model (Figure 3c). However, for the polymeric nature of the cytoskeleton, we replace springs for cables and reference the corresponding element as *cable-Maxwell*. Cables behave like springs in the extension regime, but do not resist compression. This represents the polymeric nature of the cytoskeleton, where filaments can depolymerize, buckle, or slide along each other under compressional stress. To account for the viscous environment of the cytoplasm, we additionally couple each vertex of the network to the environment via a viscous Stokes drag element.

The situation is markedly different for SFs, which are clearly visible in our fluorescence microscopy images. Here, many actin filaments are tightly bundled and cross-linked, resulting in a much more stable structure on the time scale of a few minutes up to an hour. In the model, SFs are embedded into the triangular network as a connected series of edges. As they are also known to show both viscous and elastic properties, we use the same cable-Maxwell material model for their description, though the material parameters are chosen differently to address their increased stability.

Before we describe the details of the algorithm, we want to make some remarks on the definition of the dashpot model. From a single discrete viscous element, one might expect a constant velocity response to an external force, regardless of its length, like the velocity of a sphere being dragged through a viscous fluid in a tube at constant force against Stokes' friction does not depend on the tube's length. We will find a distinctly different behavior for the dashpot, however. Its constitutive relation is defined as

$$\sigma = \eta \cdot \dot{\epsilon} \#(2.1)$$

with stress σ, dynamic viscosity η, and strain rate $\dot{\epsilon}$, which we substitute into the equation of motion,

$$\rho \frac{\partial^2 u}{\partial t^2} = \frac{\partial \sigma}{\partial x} \, , \#(2.2)$$

where u is the displacement, ρ the mass density, and x the spatial coordinate, to get

$$\frac{\partial v}{\partial t} = \frac{\eta}{\rho} \frac{\partial^2 v}{\partial x^2} \, . \#(2.3)$$

Here we substituted in the velocity $v = \partial u / \partial t$. This diffusion equation can now be solved for the boundary conditions $\sigma(a) = \sigma(b) = \eta\dot{\epsilon} = \eta \, \partial v / \partial x = f$ and $v\big((a+b)/2\big) = 0$, where a and b denote the borders of the dashpot and f is an external stress. The stationary solution found by setting $\partial v / \partial t = 0$, reads

$$v(x) = \frac{f}{\eta}\left(x - \frac{a+b}{2}\right) \, . \#(2.4)$$

Clearly, the velocity with which the endpoints move away from each other, $v_e = v(a) - v(b) = f/\eta \cdot (a-b)$, grows linearly with the system's size. A constant force velocity relation therefore only exists if one artificially holds the system size constant. In contrast, if we identify the term $a - b$ as the system size l and v_e as its time derivative, we arrive at

$$\dot{l} = \frac{f}{\eta} \cdot l \, , \#(2.5)$$

which obviously leads to an exponentially growing flow velocity of the dashpot model. Note that the last conclusion is only valid if the steady state in the diffusion equation 2.3 is reached sufficiently fast. That holds true for high velocity diffusion constants $D_v = \eta/\rho$.

This is consistent with the picture of a fixed line density λ of elements showing Stokes drag viscosity. The velocity of a single Stokes drag element is

$$v = \alpha \cdot F/\eta \, , \#(2.6)$$

where α is a proportionality constant. Coupled in series, all elements contribute to the total velocity

$$\dot{l} = v_{\mathrm{tot}} = n \cdot v = \lambda l \cdot \alpha \frac{F}{\eta} = \frac{F}{\tilde{\eta}} \cdot l \, \#(2.7)$$

with effective viscosity $\tilde{\eta}$. Note the Equations 2.5 and 2.7 are equivalent.

These findings match the expectations of a viscous polymeric system. If a piece of filamentous network

of length l flows with velocity v in response to a force f, this is due to molecular processes like the unbinding and binding of crosslinker proteins, or polymerization. If the system size is doubled to $2 \cdot l$, the number of crosslinkers, binding sites, etc. is also twice as large, resulting in a flow velocity of $2 \cdot v$. In contrast, the viscous coupling of the vertices to the environment should not depend on any distance from an arbitrary initial position, but resemble the situation of Stokes' drag.

Though the viscous environment coupling does not depend on the initial position, it has to depend on the mesh size. We expect it to be the same regardless if an area A is triangulated with n or rather $2n$ vertices, for example. Further, the viscosity should remain constant over the whole triangulated region even if the vertex density is significantly higher in some regions as in others. We therefore set $\alpha = \alpha_i = \tilde{l}/A_i$ in Eq. 2.6, where $\tilde{l}$ is a characteristic length scale, and A_i the area associated with vertex i (one third of all triangles adjacent to the vertex).

Algorithm

In order to solve the equations of motion for the network, we apply an Euler algorithm that consists of repeating the following steps:

1. Determine the total contractile force F_ν on each link ν and its elastic part F_ν^e from the current cable configuration alone.
2. Determine the force $\vec{F}_i$ on each vertex i as $\sum_\nu F_\nu \cdot \vec{e}_\nu$, where the sum runs over all the adjacent links and the $\vec{e}_\nu$ denote unit vectors along the links pointing away from v.
3. Move each vertex v by $\vec{s} = \left(\vec{F}_i \cdot \tilde{l} \cdot \Delta t\right)/(\eta^* \cdot A_i)$, where Δt is the chosen time step, η^* the viscosity of the surrounding medium, $\tilde{l}$ is a characteristic friction length scale, and A_i is the area associated with vertex i.
4. Account for the flow in the dashpot by adjusting the rest length $l_{0,\nu}$ of each cable in a cable-Maxwell element by

$$l_{0,\nu,\mathrm{new}} = l_{0,\nu} + v \cdot \Delta t = l_{0,\nu} \cdot \left(1 + \frac{F_\nu^e}{\eta_\nu A_0} \cdot \Delta t\right), \#(2.8)$$

   where Δt is the same time step as above, A_0 is the cross section of the fiber and η_ν the viscosity of the cable-Maxwell element. Note that $l_{0,\nu}$ can never decrease in a cable-Maxwell model due

to the absence of pushing forces. The rest lengths of pure cable links representing SFs are not adjusted.

5. Adjust the triangular mesh to retain a good discretization (see below).

Mesh Alterations

The description of a viscously flowing matter with a triangular mesh faces some challenges regarding mesh integrity. As discussed before, we expect sources and sinks of cytoskeletal material due to polymerization and depolymerization processes as well as compression and stretching. When we consider a source, we expect all vertices of the surrounding mesh to flow away from it. This results in increasingly large and pointed triangles in this area, until the material cannot be described with the desired resolution anymore and discretization artifacts become visible in the simulations. The converse holds for sinks: as all vertices flow inwards, triangle sizes decrease until numerical difficulties arise. As a consequence, we need to dynamically alter the mesh configuration by refining large triangles in source regions and removing small triangles at sinks. This necessarily comes at the cost that one destroys information contained in removed triangles, edges and vertices, or introduces artificial information for newly created mesh elements. Still one can retain most of the information of the original state, and we will show how to achieve this in the following sections.

To determine when mesh alterations are necessary, we define a characteristic link length $\bar{l}_0$ as the average of all resting lengths at the beginning of the simulation and a scale factor S. Then $\bar{l}_0 \cdot S$ constitutes an upper threshold for edge resting lengths, and $\bar{l}_0/S$ a lower threshold.

Triangle refinement

There are multiple ways to introduce new triangles into a triangular mesh. The first one that might come to one's mind is to replace one large triangle by four smaller ones of equal size (Supplementary Note Figure 7A). However, this introduces hanging nodes, if adjacent triangles are not refined as well. We therefore stick to an even simpler way to insert new triangles into the mesh, as shown in Supplementary Note Figure 7B. An edge connecting two triangles is split in half, if its length exceeds $\bar{l}_0 \cdot S$. The midpoint is connected to the two opposing tips of the triangles that shared the split edge. In

this way, we introduce only two new triangles in one refinement step and preserve mesh integrity.

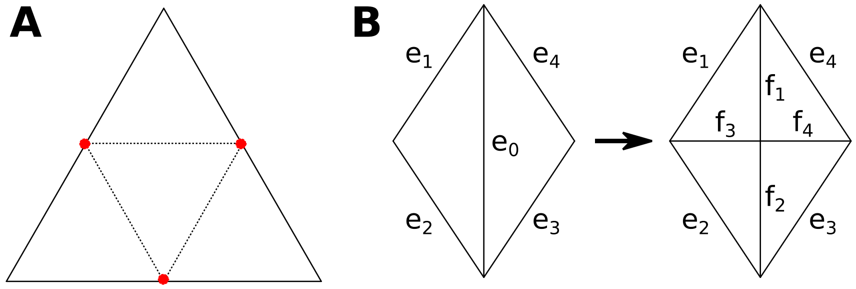

Supplementary Note Figure 7: Triangle refinement. (**A**) Triangle refinement by splitting all edges yields three additional triangles but introduces hanging nodes (red). (**B**) Refining one long edge connecting two triangles. Only two additional triangles are created without hanging nodes. Edges are denoted by e_i prior to the refinement, and f_i afterwards.

For triangle refinement it is also straightforward to define the viscous and elastic properties on the new edges. We are guided by the idea that we want to describe the same material with a different discretization, which in particular means that the forces on any vertex should not change and that the elastic properties of the links need to be adjusted. It is well known that two springs with spring constants k_0 in series behave like one spring with $k_1 = k_0/2$. So $k_{f_1} = k_{f_2} = 2k_{e_0}$ holds to describe the same material. In our simulations we will choose the spring constants for f_3, f_4 from a homogeneous background stiffness, which is set to represent the elasticity of the unstrained cytoskeleton. Regarding the viscous properties, $\eta(f_1) = \eta(f_2) = \eta(e_0)$ can be directly transferred from e_0 to f_1, f_2, since the viscosity is already defined per length. Similar to the spring constant, we choose the filament viscosity from a background viscosity for the newly created edges.

Triangle Removal

As for refinement, triangles can be removed from a mesh in many different ways. The minimal well known approach is the edge collapse algorithm, where an edge is replaced by a vertex, which is then connected to the surrounding vertices[12]. This technique is most appropriate if triangles contain one edge which is significantly shorter than the other two. In our simulations with flowing cable networks, we also observe another way of triangle shrinkage: when one height of the triangle becomes small compared to the corresponding edge. These two cases are best treated with the two procedures described below (Supplementary Note Figure 8).

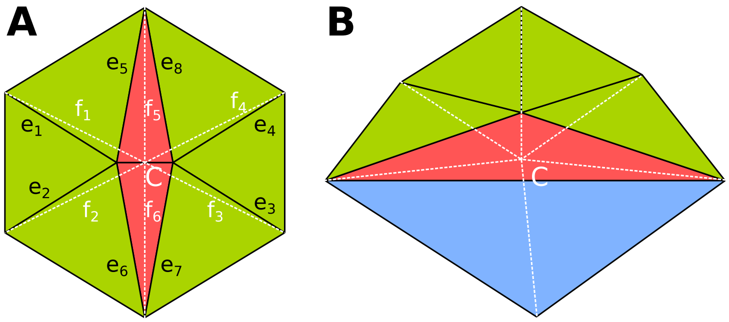

Supplementary Note Figure 8: Triangle removal. (**A**) The short edge shared by the red triangles is collapsed, only the green triangles remain. (**B**) The height of the red triangle is small compared to its longest edge and is collapsed. At the same time, the blue triangle has to be split in half to avoid hanging nods, so the overall number of triangles in the mesh is constant.

Edge Collapse

This is the standard case for triangle removal in our simulations, as it is the simplest way to reduce the overall number of triangles. The procedure is triggered if an edge becomes shorter than $\bar{l}_0/S$ and works as follows. First, the center of the short edge shared by the red triangles in Supplementary Note Figure 8A is obtained. Second, the red triangles are removed from the mesh. Third, the vertices where e_1, e_2 and e_3, e_4 meet are displaced to the center determined previously. In the last step, each of the two edges that a red triangle shared with green ones have to be merged.

Note that there are mesh configurations, in which the procedure as described above does not yield the desired results. We list here the three checks we perform before the operation is executed. If any of the checks is positive, the operation is prohibited.

1. One of the dashed white lines may intersect with one of the outer edges of a green triangle. In this case, the application of the edge collapse algorithm would lead to a failure in the mesh (some area is covered by two triangles, while some other area is uncovered at the same time).
2. Either of the red triangles in Supplementary Note Figure 8A may possess an internal structure of even smaller triangles. In this case, the algorithm would transform the sub-triangles into a straight line, which leads to undefined behavior.
3. One of the dashed white lines may become longer than a threshold for triangle refinement and thereby cause an infinite sequence of refinement and removal steps.

Height collapse

Edge collapse is not sufficient as the only way to remove triangles from the mesh. Each edge length might still be above a given threshold for a long and pointed triangle, but the triangle area can still tend towards zero. For this reason, we introduce another operation, which removes one triangle and splits another. Consequently, the overall number of triangles is conserved under this operation. However, it leads to more homogeneous distributions of triangle areas.

The procedure is triggered if the height of a triangle becomes smaller than $\bar{l}_0/(2S)$. As depicted in Supplementary Note Figure 8B, the central triangle (red) is removed, and the joint vertex of all green triangles is moved to the center of the red triangle. The blue triangle that shares the long edge of the red one is split in two in order to avoid hanging nodes. Note that the long edge is therefore split in two, and each of the parts is merged with one edge of the now neighboring green triangle.

Again, one has to check for several pitfalls that may occur for certain mesh configurations. If any of the following checks is positive, the operation is prohibited. The triangle colors now refer to the ones in Supplementary Note Figure 8B.

1. As for the edge collapse case, one of the dashed white lines may intersect with one of the outer edges of a green triangle. In this case, the application of the edge collapse algorithm would lead to a failure in the mesh (some area is covered by two triangles, while some other area is uncovered at the same time).
2. The four triangles that share an edge with the red triangle could match the height collapse criterion as well (the blue one counts as two once it is split). If they do, this can lead to an infinite chain of refinements.

Conserving important structures during triangle removal

Note that we introduce one new vertex in each of the triangle removal operations. Its position is indicated in Supplementary Note Figure 8 by the point where the dotted white lines merge. However, this might not be appropriate if the operations are to be performed in the presence of fixed vertices, free boundaries, or stress fibers. These structures are to be preserved, as they are of special

importance to the simulations. This means for fixed vertices to keep their position, and for edges at the boundary or edges of stress fibers to remain straight lines. If it is not possible to retain all structures unperturbed, we give highest priority to keeping the location of fixed vertices, followed by vertex locations at the boundary and within stress fibers in this order.

Cable properties during triangle removal

We are again guided by the idea to describe the same material, now with a coarser discretization. Forces on vertices and local elastic properties should not change. However, it is not possible to achieve this completely, since we are removing information from the mesh. In the edge collapse case, the orientation and therefore the force direction of edges $e_1 - e_4$ in Supplementary Note Figure 8A changes only slightly, since the collapsed edge is short. However, force magnitudes may change significantly depending on the local spring constants. We therefore adjust the resting length for each of these edges such that $F(e_i) = F(f_i)$, where F denotes the edge force, while the spring constants are kept constant. As e_6, e_7 and e_5, e_8 are merged, their spring constants k are added to yield $k(f_5) = k(e_5) + k(e_8)$ and $k(f_6) = k(e_6) + k(e_7)$. In this sum we account only for edges that are in the extension limit, as cables cannot build up compression forces. Rest lengths l_0 are chosen such that the force of the merged edge equals the sum of its two predecessors. If both preceding edges do not bear force, the rest length is ill-defined because of the flat force-extension curve for cables in the compression regime. In this case, we choose the new rest length such that the length difference Δl between current length l and rest length l_0, $\Delta l = l - l_0$, of the merged edge is the average of the two predecessors. If the preceding edges carry active forces, the new edge will carry the summed force.

In the height collapse case, we combine the algorithms for edge splitting and edge merging, that are adopted from the edge collapse and the triangle refinement operations. The edge shared by the red and the blue triangle is split as in the triangle insertion section, and each of the two parts is merged with the corresponding edge share by the red and a green triangle.

Regarding the viscous properties, we note that splitting an edge does not change the filament viscosity as it is defined per length (see above). Merging however does increase the viscosity of the single

dashpots to their sum in the joint element. So for merging, filament viscosities are treated just as it is explained above for the spring constants.

Edge flipping

After some repetitions of triangle insertion and removal, some vertices show an increase in connectivity, i.e. in the number of edges connected to one vertex, which ultimately leads to numerical and algorithmic difficulties (e. g. the problems discussed in the triangle removal section). We introduce edge flipping in order to keep a homogeneous distribution of connectivity. If two triangles are connected by an edge e, and if the two triangle angles opposite to e are larger than a threshold angle $\alpha > \pi/2$, the edge is flipped and connects now the two vertices which where unconnected before. During this operation, we can conserve hardly any elastic properties of the material. We simply select the spring constant for the new edge from the background elasticity and apply zero strain. To keep these events rare, we use $\alpha = 110^{\circ}$ in our simulations.

Active contraction

To induce the active contraction of the mesh, we assign force dipoles F_{ν} to edges ν in the activated region of the cell (Figure 3c). Further, if the force dipoles are assigned to both SF and background network edges, and the contractile zone does not span the whole width of the cell, SFs are indented towards the center. As this does not occur in experiments, we conclude that SFs are the only relevant active elements (Figure 3e,f) and restrict the assignment to SF edges. As in the one-dimensional model, we ramp the force level up and down with an exponentially plateauing function, $F = F_0\big(1 - \exp(-t/\tau)\big)$, where tau is a time constant fitted to be $\tau \approx 70s$ with the continuum model.

Parameter Choice

In order to compare the discrete model to the continuum model, we derive the main parameters from the continuum quantities according to Caillerie *et al.*[13]. For this purpose, we set the spring constant to $k_{\mathrm{SF}} = \sqrt{3}/2hE_{\mathrm{c}}$, where $h = 1\ \mu m$ is the system height and E_c is the Young's modulus from the continuum model. Likewise, we evaluate $\eta_{\mathrm{SF}} = \sqrt{3}/2h\eta_{\mathrm{c}}$ and $\eta_{\mathrm{SF}}/\gamma = \eta_{\mathrm{c}}/\gamma_{\mathrm{c}}$. For the fitting of the kymograph shown in Figure 4h, we used the fitted parameters from the continuum model, which are

$E_c = 2.3\ kPa$, $\eta_c = 6.8\ MPa \cdot s$, and $\gamma_c = 0.03\ kg \cdot s/\mu m^3$.

Supplementary References

1. Prost, J., Julicher, F. & Joanny, J.-F. Active gel physics. *Nature Physics* **11,** 111–117 (2015).
2. Tlili, S. *et al.* Colloquium: Mechanical formalisms for tissue dynamics. *Eur Phys J E Soft Matter* **38,** 121–31 (2015).
3. Hill, A. V. The Mechanical Efficiency of Frog's Muscle. in **127,** 434–451 (1939).
4. Besser, A., Colombelli, J., Stelzer, E. H. K. & Schwarz, U. S. Viscoelastic response of contractile filament bundles. *Phys Rev E* **83,** 051902 (2011).
5. Edwards, C. M. & Schwarz, U. S. Force localization in contracting cell layers. *Phys Rev Lett* **107,** 128101 (2011).
6. Oakes, P. W., Beckham, Y., Stricker, J. & Gardel, M. L. Tension is required but not sufficient for focal adhesion maturation without a stress fiber template. *J Cell Biol* **196,** 363–374 (2012).
7. Alnæs, M. *et al.* The FEniCS Project Version 1.5. *Archive of Numerical Software* **3,** 9–23 (2015).
8. Banerjee, S. & Marchetti, M. C. Contractile stresses in cohesive cell layers on finite-thickness substrates. *Phys Rev Lett* **109,** 108101 (2012).
9. Nelder, J. A. & Mead, R. A Simplex Method for Function Minimization. *The Computer Journal* **7,** 308–313 (1965).
10. Étienne, J. *et al.* Cells as liquid motors: mechanosensitivity emerges from collective dynamics of actomyosin cortex. *Proc. Natl. Acad. Sci. U.S.A.* **112,** 2740–2745 (2015).
11. Saha, A. *et al.* Determining Physical Properties of the Cell Cortex. *Biophys J* **110,** 1421–1429 (2016).
12. Lindstrom, P. & Turk, G. Fast and memory efficient polygonal simplification. in 279–286, (IEEE, 1998). doi:10.1109/VISUAL.1998.745314
13. Caillerie, D., Mourad, A. & Raoult, A. Discrete Homogenization in Graphene Sheet Modeling. *J Elasticity* **84,** 33–68 (2006).
